# Difference of the associations between self-rated health and demographic characteristics, lifestyle, and psychosocial work environment between two types of Chinese worksite

**DOI:** 10.1186/1471-2458-14-851

**Published:** 2014-08-15

**Authors:** Yingnan Jia, Junling Gao, Junming Dai, Pinpin Zheng, Xiaoyu Wu, Guangyao Li, Hua Fu

**Affiliations:** School of Public Health, Health Communication Institute, Fudan University, Shanghai, 200032 China; Shanghai Municipal Health Promotion Committee Office, Shanghai, 200002 China

**Keywords:** Self-rated health, Demographic characteristics, Lifestyle, Psychosocial work environment, Type of worksite

## Abstract

**Background:**

Although studies of self-rated health (SRH) are conducted widely in developed countries, comprehensive assessments of the determinants of SRH in Chinese are scarce, particularly for working Chinese individuals. Determinants of SRH might differ among worksites based on differences in the nature and stress associated with different jobs, work intensity, and the lifestyles of employees.

**Methods:**

Two thousand and forty questionnaires that addressed SRH, demographic characteristics, lifestyle, and the psychosocial work environment were administered to employees at two worksites. A total of 1644 subjects provided complete data for analysis (80.6% response rate).

**Results:**

Participants from government departments had significantly better SRH than did those from high-tech enterprises (61.1% vs. 67.5%, respectively). Lifestyles were significantly less healthy at government departments compared with high-tech enterprises, whereas the psychosocial work environment was better. The results of unadjusted and adjusted models revealed differences between the potential health-influencing factors of participants based on their type of worksite. In logistic regression models, gender was strongly associated with SRH in all participants, whereas length of service was correlated with SRH only in participants from high-tech enterprises. In high-tech enterprises, good SRH was less common in physically inactive subjects vs. physically active participants (OR = 0.561). In government departments, passive smoking was negatively associated with SRH significantly. Social capital (OR = 1.073) and job control (OR = 1.550) were positively correlated with SRH in high-tech enterprises. Job control was the only psychosocial factor significantly associated with SRH in government departments.

**Conclusions:**

Participants from different types of worksite reported different SRH, healthy lifestyles, and psychosocial work environments. Moreover, the association between SRH and demographic characteristics, lifestyle, and the psychosocial work environment significantly differed by type of worksite.

## Background

SRH is generally assessed by a single item index or a single survey question inviting respondents to provide a subjective assessment of their health using some form of a five-point scale [1]. In a wide variety of populations, SRH is one of the most widely used methods to assess the public health status of adult populations, and is associated with objective health indices including physical and functional health, as well as physician’s ratings [2]. This indicator has become increasingly popular for assessing health status because of its simplicity and solid well-established links with various health indicators such as mortality [3–6] and chronic diseases [7, 8].

The prevalence of chronic diseases such as cardiovascular disease and diabetes has been increasing rapidly in recent years along with recent economic development [9], suggesting that modern work habits and unhealthy lifestyles are threatening the health of the Chinese population. Due to the fast pace of life and job pressure, health problems have become increasingly prominent in Chinese employees. Some previous studies confirmed that the poor health of working people is a major risk factor for early retirement [10, 11]. However, comprehensive assessments of the determinants of SRH among Chinese populations are rare, particularly in working individuals. Previous studies evaluated the levels of SRH among Chinese subjects, but they considered only a single demographic group and lifestyle factor, ignoring the potential effect of psychosocial work environments [12–15].

Compared with studies of Chinese populations, those conducted in developed countries indicated more valuable and in-depth results. Some studies on the determinants of SRH revealed that positive health-related lifestyles might have a positive effect [16], and that health-related behaviors such as smoking and alcohol intake are associated with poor SRH [17]. Important associations were identified between physical activity and good SRH after adjusting for demographic and other lifestyle factors [18, 19]. In addition to lifestyle factors, the psychosocial work environment such as social capital (features of social relationships that facilitate collective action for mutual benefit), job demand (the need to work quickly and hard), job control (lack of control over skill use, time allocation, and organizational decisions), and support at work (social support from leadership and colleagues) were also associated with SRH [20–22]. Moreover, a study revealed that employee health could vary in different sized enterprises [23]. Due to differences in job content, job stress, work intensity, and employee lifestyles at various types of worksite, the potential determinants of SRH might also differ according to the type of worksite. However, the effect of type of worksite on the association between SRH and lifestyle and psychosocial factors remains unclear. To develop more effective and targeted interventions, it is important to define the roles played by lifestyle and psychosocial factors in health disparities.

In this study, we assessed participants from two representative types of worksite, including 10 government departments and two high-tech enterprises. These types of worksite have comparable features such as a relatively stable time arrangement, mental labor, and high education requirement. However, high-tech enterprises were perceived as high job demand and fast work rhythm because employees had to update their specialized knowledge to accomplish sophisticated duties on time without error. In contrast, civil servants are considered to have the most stable job in China. Most civil servants are responsible for drawing up plans and providing guidance and supervision for relevant activities. Although civil servants are perceived to have an easy job, they have inflexible schedules and a lack of decision-making ability.

The aim of this study was to investigate and compare the association between SRH and demographic characteristics (gender, age, education, marital status, and length of service), lifestyle (smoking, alcohol intake, physical activity, and passive smoking), and psychosocial work environment (social capital, job demand, job control, and support at work) between participants from two representative types of Chinese worksite.

## Methods

### Informed consent form

Written informed consent statement forms were obtained from respondents, and the right to withdraw and autonomy of the responses were explained. The study received ethical approval from the ethics committee of School of Public Health of Fudan University, China.

### Data collection

Twelve interviewers underwent a training lesson in which the standard guidelines for the investigation were emphasized. Each interviewer was responsible for collecting the self-administered questionnaires from each worksite during the working day from July to September 2012.

### Participants

In this study, we assessed participants at two representative types of worksite. The participants consisted of 2040 employees who worked in Shanghai. One thousand, six hundred and forty-four subjects provided complete data for analysis, which was a response rate of 80.6%. Participants were recruited from 12 different worksites: 10 government departments and two high-tech enterprises.

### Demographic variables

Self-reported demographic variables included gender, age, education, length of service, and marital status. Age and length of service were separated into four and five categories, respectively. The four age categories were <30, 30 - 39, 40 - 49, and >50 years. The five categories for length of service were <5, 5 - 9, 10 - 14, 15 - 19, and ≥20 years.

### Measurements

#### SRH

SRH was the main health outcome of this study. Respondents were asked to rate their own general health on a five-point scale ranging from perfect to poor. As the dependent variable in logistic regression, the original variable was dichotomized according to the distribution of SRH, with 1 representing perfect, very good, and good health, and 0 representing fair and poor health.

#### Health behavior

Four health behaviors and two potential influencing factors were included in the study. Current smoking status (current smoker, ex-smoker, or non-smoker), passive smoking (yes/no), alcohol intake (weekly, monthly, or never), and physical activity (high, moderate, or low) were assessed using self-reporting questionnaires. People who had smoked more than 100 cigarettes were defined as smokers. Passive smokers were respondents who had been exposed to others’ smoke for more than 15 minutes in the last week. The respondents who had drunk alcohol were defined as alcohol drinkers. The self-reported data for physical activity were collected from the Chinese version short IPAQ (International Physical Activity Questionnaire), which was acceptably reliable (ICC of 0.79 and %CV of 26%) [24].

The IPAQ short form asked about three specific types of physical activity (PA) including walking, moderate-intensity activities such as dancing, cycling, and performing tai chi, and vigorous-intensity activities such as swimming and playing basketball. The following values were used to analyze IPAQ data: walking = 3.3 metabolism equivalents (METs; moderate PA = 4.0 METs, and vigorous PA = 8.0 METs). Calculating of the total score for the short form required summation of the duration (in minutes) and frequency (days) of walking, moderate-intensity activities, and vigorous-intensity activities. Three levels of physical activity were proposed to classify the populations: low, moderate, and high. The two criteria for classification as “high” were (a) vigorous-intensity activity on at least 3 days achieving a minimum total physical activity of 1500 MET-minutes per week, or (b) 7 or more days of any combination of walking, moderate-intensity, or vigorous-intensity activities achieving a minimum total physical activity of 3000 MET-minutes per week. The pattern of activity classified as “moderate” was either of the following criteria: (a) 3 or more days of vigorous-intensity activity of at least 20 minutes per day, (b) 5 or more days of moderate-intensity activity and/or walking for at least 30 minutes per day, or (c) 5 or more days of any combination of walking, moderate-intensity, or vigorous intensity activities achieving a minimum total physical activity of 600 MET-minutes per week. Individuals who did not meet the criteria for “moderate” or “high” were considered to have a “low” level of physical activity.

#### Psychosocial work environment

The social capital scale measured both the cognitive and structural components of social capital using eight items [25]. The internal consistency of the scale was good (Cronbach’s alpha = 0.88) [25]. The eight Likert-scale items (range of scale: 1 - 5) are presented in Table 1. A summary score of the ratings of all social capital items was constructed, in which a high score indicated high social capital. For the eight-item measure of social capital, the internal consistency of the scale was good (Cronbach’s alpha = 0.88). Questions regarding job demand, job control, and support at work were based on a Karasek’s Job Content Instrument with Cronbach’s alpha (0.88) [26, 27]. Five factors were used to measure the psychological demand of the job: working very carefully, working very fast, using a lot of information, freedom from conflicting demands, and requests to do an excessive amount of work. Decision latitude was assessed using four questions about the employee’s ability to use and develop skills (by asking if the job involves learning new things, non-repetitive work, creativity, and a high skill level) and exert authority (by assessing freedom to decide how to perform work, and the ability to make one’s own decisions). Professional support was assessed by four questions regarding support from colleagues, supervisors, family, and workplace. All Likert-scale items are presented in Table 1.

**Table 1:** **Work-related items in the questionnaire**

Variable	Item
Social capital	1. Our supervisor treats us with kindness and consideration.
	2. Our supervisor shows concern for our rights as an employee.
	3. We have a “we are together” attitude.
	4. People keep each other informed about work-related issues in the work unit.
	5. People feel understood and accepted by each other.
	6. Do members of the work unit build on each other’s ideas to achieve the best possible outcome?
	7. People in the work unit co-operate to help develop and apply new ideas.
	8. We can trust our supervisor.
Job demand	1. My job requires me to work very carefully.
	2. My job requires me to do an excessive amount of work.
	3. My job requires me to use a lot of information.
	4. My job requires me to work very fast.
	5. I have freedom from conflicting demands.
Job control	1. I have freedom to decide how to perform work.
	2. I have the ability to make decisions on my own.
	3. My job involves learning new things.
	4. A high level of skill is needed.
	5. My job requires me to be creative.
	6. My job requires me to do non-repetitive work.
Support at work	1. My colleagues support my job.
	2. My supervisors support my job.
	3. My family supports my job.
	4. My workplace supports my job.

For social capital questions, 1 = fully disagree, indicative of low social capital; 5 = fully agree, indicative of high social capital. For job demand questions, 1 = fully disagree, indicative of low job demand; 5 = fully agree, indicative of high job demand. For job control questions, 1 = fully disagree, indicative of low job control; 5 = fully agree, indicative of high job control. For support at work, 1 = fully disagree, indicative of low support; 5 = fully agree; indicative of high support.

### Data analyses

Participants were classified into two groups according to the type of worksite: high-tech enterprise employees and government department employees. Descriptive analysis, analysis of variance, and chi-squared tests were conducted to compare the differences between SRH and potential health-influencing factors between the two groups.

Multiple logistic regression analysis was conducted to examine the factors that influenced SRH among participants from different types of worksite. In either group, a binomial logistic regression analysis was performed in three steps following the theoretical framework presented in the Introduction. First, background variables (gender, age, education, marital status, and length of service) were included in models A and B. Next, lifestyle factors (physical activity, smoking, alcohol intake, passive smoking) were added to models C and D. Finally, psychosocial work environment factors (social capital, job demand, job control, and social support at work) were introduced into models E and F. Unadjusted and multivariate-adjusted odds ratios (OR) and 95% confidence intervals (CI) were calculated from logistic regressions to examine the factors that influence SRH. The 2log-liklihood and significance of the Hosmer-Lemeshow test were also reported on each model to allow us to evaluate the adequacy of changes in the model and additional variables. For all analyses, statistical significance was set at 0.05. Statistical analysis was performed using the Statistical Package for Social Sciences (IBM SPSS 20).

## Results

### Participant characteristics

Data were collected from 2,040 participants from 12 different worksites comprising 10 government departments and two high-tech enterprises. The demographics of the study sample are reported in Table 2. The final sample consisted of 1,644 participants (59.5% male and 40.5% female). There were significant differences in the distribution of all the demographic characteristics including gender, age, length of service, marital status, and education between high-tech enterprises and government departments. The proportion of male participants working in high-tech enterprises was higher than in government departments (χ^2^ = 22.125, *P* < 0.001). The distribution of age differed by type of work. In high-tech enterprises, nearly half of the participants were <30 years of age, compared with only 17.2% in the government departments (χ^2^ = 219.398, *P* < 0.001). The proportions of all other age groups were similar. The distribution of length of service had similar characteristics as age (χ^2^ = 106.845, *P* < 0.001). A total of 62.5% of participants from high-tech enterprises had been married, compared with 90.3% of government workers. More than half of the respondents had a bachelor’s or higher degree in both groups.

**Table 2:** **Demographics of the participants by types of work**

Variables		Overall ***n***(%)	High-tech enterprise ***n***(%)	Government department ***n***(%)	χ ^2^ ( ***P***)	Fair or poor SRH ***n***(%)	Good SRH ***n***(%)	χ ^2^ ( ***P***)
Gender	Male	978 (59.5)	488 (65.8)	490 (54.3)	22.125 (<0.001)	314 (32.1)	664 (67.9)	11.462 (0.001)
	Female	666 (40.5)	254 (34.2)	412 (45.7)		268 (40.2)	398 (59.8)	
Age, years	~30	517 (31.4)	362 (48.8)	155 (17.2)	219.398 (<0.001)	150 (29.0)	367 (71.0)	14.897 (0.002)
	30~	484 (29.4)	209 (28.2)	275 (30.5)		176 (36.4)	308 (63.6)	
	40~	350 (21.3)	91 (12.3)	259 (28.7)		139 (39.7)	211 (60.3)	
	50~	293 (17.8)	80 (10.8)	213 (23.6)		117 (39.9)	176 (60.1)	
Length of service, years	~5	625 (38.0)	365 (49.2)	260 (28.8)	106.845 (<0.001)	180 (28.8)	445 (71.2)	27.567 (<0.001)
	5~	371 (22.6)	112 (15.1)	259 (28.7)		128 (34.5)	243 (65.5)	
	10~	240 (14.6)	85 (11.5)	155 (17.2)		93 (38.8)	147 (61.3)	
	15~	136 (8.3)	39 (5.3)	97 (10.8)		61 (44.9)	75 (55.1)	
	20~	272 (16.5)	141 (19.0)	131 (14.5)		120 (44.1)	152 (55.9)	
Marital status	Married	1261 (76.7)	447 (60.2)	814 (90.2)	205.059 (<0.001)	470 (37.3)	791 (62.7)	8.282 (0.004)
	Unmarried/Divorced/Widowed	383 (23.3)	295 (39.8)	88 (9.8)		112 (29.2)	271 (70.8)	
Education	Junior high school	109 (6.6)	45 (6.1)	64 (7.1)	147.215 (<0.001)	41 (37.6)	68 (62.4)	23.490 (<0.001)
	High School/Technical secondary school	266 (16.2)	162 (21.8)	104 (11.5)		110 (41.4)	156 (58.6)	
	Junior college	325 (19.8)	100 (13.5)	225 (24.9)		138 (42.5)	187 (57.5)	
	Bachelor’s degree	772 (47.0)	299 (40.3)	473 (52.4)		228 (29.5)	544 (70.5)	
	Master’s degree/Doctorate	172 (10.5)	136 (18.3)	36 (4.0)		65 (37.8)	107 (62.2)	

Table 2 shows that all the demographic characteristics were significantly associated with SRH. The proportion of male participants who reported good SRH was higher than that of female participants (χ2 = 11.462, *P* = 0.001). The proportion of individuals who reported good SRH decreased significantly with age (χ^2^ = 14.897, *P* = 0.002). Similar characteristics were found among the different groups of length of service (χ^2^ = 27.567, *P* < 0.001). The proportion of married participants who reported good SRH was lower than that of the unmarried participants (χ^2^ = 8.282, *P* = 0.004). The proportion of individuals with a bachelor’s degree who reported good SRH was higher than the other groups (χ^2^ = 23.490, *P* < 0.001).

### Difference of potential health influencing factors of participants from different types of worksite

Table 3 shows the differences in health and potential health-influencing factors between the two work groups. Participants from government departments had significantly better SRH (67.5%) compared with those from high-tech enterprises (61.1%).

**Table 3:** **Comparison of health and potential health influencing factors of participants by types of work**

	Overall ***n***(%)	High-tech enterprise ***n***(%)	Government department ***n***(%)	F(P)/χ ^2^(P)	Good SRH ***n***(%)	Fair or poor SRH ***n***(%)	F (P)/χ ^2^ (P)
SRH				7.441 (0.006)			/
Good	1062 (64.6)	453 (61.1)	609 (67.5)		-	-	
Fair and bad	582 (35.4)	289 (38.9)	293 (32.5)		-	-	
Physical activity				20.261 (<0.001)			18.446 (<0.001)
Low	575 (35.0)	226 (30.5)	349 (38.7)		243 (42.3)	332 (57.7)	
Moderate	796 (48.4)	364 (49.1)	432 (47.9)		249 (31.3)	547 (68.7)	
High	273 (16.6)	152 (20.5)	121 (13.4)		90 (33.0)	183 (67.0)	
Smoking				28.345 (<0.001)			0.310 (0.856)
Current	431 (26.2)	148 (19.9)	283 (31.4)		157 (36.4)	274 (63.6)	
Ex-smoker	86 (5.2)	38 (5.1)	48 (5.3)		31 (36.0)	55 (64.0)	
Never smoked	1127 (68.6)	556 (74.9)	571 (63.3)		394 (35.0)	733 (65.0)	
Alcohol intake				64.601 (<0.001)			7.216 (0.027)
Weekly	230 (14.0)	50 (6.7)	180 (20.0)		77 (33.5)	153 (66.5)	
Monthly	511 (31.1)	229 (30.9)	282 (31.3)		160 (31.3)	351 (68.7)	
Never	903 (54.9)	463 (62.4)	440 (48.8)		345 (38.2)	558 (61.8)	
Passive smoking				187.550 (<0.001)			6.014 (0.014)
Yes	922 (56.1)	279 (37.6)	643 (71.3)		350 (38.0)	572 (62.0)	
No	722 (43.9)	463 (62.4)	259 (28.7)		232 (32.1)	490 (67.9)	
Social capital	32.49 ± 5.233	30.80 ± 5.600	33.89 ± 4.452	156.056 (<0.001)	31.43 ± 0.564	33.08 ± 4.900	38.027 (<0.001)
Job demand	3.58 ± 0.570	3.77 ± 0.583	3.42 ± 0.506	175.305 (<0.001)	3.58 ± 0.575	3.58 ± 0.567	0.009 (0.925)
Job control	3.38 ± 0.581	3.38 ± 0.598	3.38 ± 0.567	0.037 (0.848)	3.26 ± 0.614	3.44 ± 0.552	36.667 (<0.001)
Support at work	4.15 ± 0.574	4.03 ± 0.607	4.26 ± 0.523	66.861 (<0.001)	4.08 ± 0.587	4.20 ± 0.562	15.901 (<0.001)

For potential health influencing factors, participants from government departments had significantly more social capital, lower job demand, and more professional support than did those from high-tech enterprises. The scores for job control were comparable. Compared with participants from high-tech enterprises, respondents from government departments led significantly unhealthier lifestyles with less physical activity, increased smoking and passive smoking rates, and more alcohol consumption.

Table 3 shows that some health behaviors were significantly associated with SRH. The proportion of physically inactive participants who reported good SRH was lower than the other participants (χ^2^ = 20.261, *P* < 0.001). A total of 68.7% of participants who drank alcohol monthly reported good SRH, which was the highest among the three groups (χ^2^ = 64.601, *P* < 0.001). The proportion of the passive smokers who reported good SRH was lower than that of non-passive smokers (χ^2^ = 187.550, *P* < 0.001). There were also significant differences between SRH in groups of all the psychosocial work environment factors, except for job demand.

### Results of logistic regression analyses

Logistic regression analyses were used to identify the potential factors that influenced SRH (Tables 4 and 5). Half of the six models were based on data from participants in high-tech enterprises (models A, C, and E), and half were from government departments (models B, D, and F).

**Table 4:** **Odds ratios (OR) and 95% confidence intervals (CI) of predictors of self-rated good health among participants from high-tech enterprises**

	Model A: ***n*** = 742 (High-tech enterprise) Good self-rated health	Model C: ***n*** = 742 (High-tech enterprise) Good self-rated health	Model E: ***n*** = 742 (High-tech enterprise) Good self-rated health
Variables	OR (95% CI)	OR (95% CI)	OR (95% CI)
Male	1.556 (1.126-2.150)**	1.544 (1.059-2.250)*	1.511 (1.027-2.223)*
Female	Reference	Reference	Reference
Age, years			
~30	0.490 (0.203–1.181)	0.512 (0.209–1.258)	0.535 (0.213–1.345)
30~	0.554 (0.244–1.260)	0.577 (0.249–1.335)	0.662 (0.277–1.579)
40~	0.441 (0.226–0.859)*	0.416 (0.208–0.833)*	0.473 (0.232–0.965)*
50~	Reference	Reference	Reference
Education			
Junior high school	2.035 (0.966–4.286)	2.463 (1.151–5.271)*	2.995 (1.361–6.592)**
High school/ Technical secondary school	1.358 (0.809–2.279)	1.526 (0.879–2.651)	2.145 (1.202–3.828)*
Junior college	0.982 (0.561–1.721)	1.103 (0.619–1.968)	1.378 (0.759–2.502)
Bachelor’s	1.362 (0.881–2.107)	1.441 (0.923–2.250)	1.475 (0.936–2.323)
Masters/Doctorate	Reference	Reference	Reference
Married	1.325 (0.879–1.996)	1.200 (0.790–1.824)	1.111 (0.724–1.704)
Unmarried/Divorced/Widowed	Reference	Reference	Reference
Length of service, years			
~5	3.113 (1.450–6.684)**	2.734 (1.252–5.971)*	2.778 (1.246–6.195)*
5~	2.109 (0.968–4.597)	1.904 (0.857–4.231)	1.891 (0.830–4.307)
10~	1.657 (0.766–3.585)	1.495 (0.678–3.295)	1.467 (0.650–3.307)
15~	1.151 (0.483–2.739)	1.024 (0.423–2.476)	1.064 (0.430–2.631)
20~	Reference	Reference	Reference
Physical activity			
Low		0.561 (0.353–0.890)*	0.553 (0.345–0.888)*
Moderate		0.971 (0.625–1.510)	0.940 (0.597–1.480)
High		Reference	Reference
Non-smoker		1.423 (0.890–2.276)	1.488 (0.916–2.416)
Ex-smoker		1.425 (0.642–3.162)	1.385 (0.607–3.161)
Current Smoker		Reference	Reference
Non-alcohol drinker		0.585 (0.299–1.147)	0.514 (0.255–1.035)
Alcohol intake (monthly)		0.754 (0.376–1.514)	0.679 (0.330–1.397)
Alcohol intake (weekly)		Reference	Reference
Passive smoker		0.730 (0.519–1.028)	0.756 (0.532–1.074)
Non-passive smoker		Reference	Reference
Social capital			1.073 (1.026–1.122)**
Job demand			0.867 (0.637–1.179)
Job control			1.550 (1.135–2.116)**
Support at work			0.829 (0.558–1.232)

Model A included only demographic variables. Model C also included physical activity, smoking, alcohol intake, and passive smoking. Model E also included social capital, job demand, job control, support at work in addition to the factors in model C. Results of -2log-liklihood: Model A, 942.969; Model C, 922.487; Model E, 891.893. Significance of Hosmer-Lemeshow test: Model A, 0.741; Model C, 0.659; Model E, 0.093. ^*^ *P* < 0.05; ^**^ *P* < 0.01.

**Table 5:** **Odds ratios (OR) and 95% confidence intervals (CI) of predictors of self-rated good health among participants from government departments**

	Model B: ***n*** = 902 (Government department) Good self-rated health	Model D: ***n*** = 902 (Government department) Good self-rated health	Model F: ***n*** = 902 (Government department) Good self-rated health
Variables	OR (95% CI)	OR (95% CI)	OR (95% CI)
Male	1.918 (1.392–2.644)**	2.546 (1.588–4.084)**	2.568 (1.598–4.126)**
Female	Reference	Reference	Reference
Age, years			
~30	2.413 (1.265–4.602)**	2.438 (1.254–4.740)**	2.635 (1.343–5.168)**
30~	1.478 (0.914–2.388)	1.464 (0.894–2.397)	1.558 (0.945–2.569)
40~	1.403 (0.930–2.116)	1.338 (0.882–2.028)	1.361 (0.893–2.073)
50~	Reference	Reference	Reference
Education			
Junior high school	0.732 (0.286–1.876)	0.792 (0.301–2.083)	0.977 (0.363–2.628)
High school/Technical secondary school	0.603 (0.252–1.444)	0.653 (0.269–1.589)	0.729 (0.297–1.785)
Junior college	0.678 (0.300–1.533)	0.706 (0.308–1.618)	0.791 (0.343–1.825)
Bachelor’s	1.259 (0.577–2.747)	1.323 (0.600–2.920)	1.367 (0.617–3.031)
Masters/Doctorate	Reference	Reference	Reference
Married	0.959 (0.536–1.717)	0.903 (0.498–1.638)	0.929 (0.510–1.691)
Unmarried/Divorced/ Widowed	Reference	Reference	Reference
Length of service, years			
~5	0.914 (0.530–1.577)	0.971 (0.557–1.692)	0.975 (0.553–1.719)
5~	0.807 (0.490–1.329)	0.800 (0.481–1.332)	0.829 (0.495–1.389)
10~	0.868 (0.519–1.451)	0.888 (0.525–1.499)	0.906 (0.533–1.542)
15~	0.746 (0.425–1.309)	0.722 (0.406–1.284)	0.752 (0.421–1.345)
20~	Reference	Reference	Reference
Physical activity		0.047	0.053
Low		0.815 (0.510–1.302)	0.842 (0.524–1.354)
Moderate		1.214 (0.759–1.943)	1.249 (0.777–2.007)
High		Reference	Reference
Non-smoker		1.286 (0.792–2.087)	1.421 (0.870–2.323)
Ex-smoker		0.855 (0.436–1.676)	0.823 (0.418–1.620)
Current smoker		Reference	Reference
Non-alcohol drinker		0.952 (0.600–1.512)	0.962 (0.603–1.533)
Alcohol intake (monthly)		1.025 (0.661–1.588)	1.020 (0.656–1.586)
Alcohol intake (weekly)		Reference	Reference
Passive smoker		0.537 (0.378–0.763)**	0.552 (0.388–0.787)**
Non-passive smoker		Reference	Reference
Social capital			1.004 (0.956–1.054)
Job demand			1.024 (0.754–1.392)
Job control			1.525 (1.132–2.056)**
Support at work			1.005 (cxx0.659–1.533)

Model B included only demographic variables. Model D also included physical activity, smoking, alcohol intake, and passive smoking. Model F also included social capital, job demand, job control, and support at work, in addition to the factors in models B and D. Results of -2log-liklihood: Model B, 1089.559; Model D, 1067.222; Model F, 1057.217. Significance of Hosmer-Lemeshow Test: Model B, 0.683; Model D, 0.795; Model F, 0.751. ^**^ *P* < 0.01.

Gender had a strong association with SRH in all participants, whereas length of service was associated with SRH in participants from high-tech enterprises. Participants employed for <5 years were more likely to have good SRH (OR = 3.113) compared with those employed for >20 years. Age and length of service were also associated with SRH in different directions. Moreover, these associations varied according to type of worksite. For participants from high-tech enterprises, people <50 years old were less likely to have good SRH (OR <0.5) than subjects >50. Consistent with this, participants who were employed for <20 years were more likely to have good SRH (OR <0.5) compared with those employed for >20 years. For participants from government departments, people <50 years old were more likely to have good SRH (OR >1.0) than respondents >50. However, individuals who were employed for <20 years were less likely to have good SRH (OR <1.0) than those employed >20 years. Although there was some variation between different subgroups of age and length of service, there were no significant differences.

Some lifestyle factors were also related to SRH after adjusting for demographic variables. For participants from high-tech enterprises, good SRH was less common (OR = 0.561) among physical inactive participants compared with highly physically high active ones. For participants from government departments, passive smoking was negatively correlated with SRH significantly. The effects of lifestyle factors were mainly independent, and did not change when psychosocial factors were introduced into the model. Adding the lifestyle factors into the model attenuated the association between length of service and SRH in participants from high-tech enterprises. However, for participants from government departments the association between gender and SRH was strengthened after the addition of lifestyle factors.

Psychosocial factors were introduced into the final models. Some psychosocial factors were associated with SRH after adjusting for demographic variables and lifestyle factors. For model E, social capital (OR = 1.073) and job control (OR = 1.550) were positively associated with SRH in participants from high-tech enterprises. For model F, job control was the only psychosocial factor significantly associated with SRH in participants from government departments. After considering psychosocial factors, the association between education and SRH was strengthened in participants from high-tech enterprises, whereas the association was attenuated in participants from government departments.

## Discussion

We found a modest level of good SRH among all participants (64.6%), which varied significantly by type of worksite. Lim et al. studied 6236 individuals aged ≥18 in Singapore (an urban area) and found a level of good or excellent SRH in 77% of respondents [15]. Similarly, Pei et al. studied 9594 Chinese subjects aged ≥18, and also found a level of good SRH of 72% [28]. The discrepancy between our study and these reports might be due to different distributions of employment. Participants from government departments had significantly improved SRH (67.5%) than those from high-tech enterprises (61.1%). Consistent with our findings, a study conducted in China also found that civil servants reported better health than scientific or technical personnel [29].

Compared with civil servants, participants from high-tech enterprises led a significantly healthier lifestyle. Specifically, they were more physically active (69.6 vs. 61.3%), smoked less (19.9 vs. 31.4%), had less alcohol intake (37.6 vs. 51.3%), and less passive smoking (37.6 vs. 71.3%). Another study showed that civil servants were more physically active than company staff (67.7 vs. 67.1%) [30]. These inconsistent findings might be due to the different characteristics of the participants. The proportion of smoking and passive smoking among civil servants in our study was comparable with other Chinese studies [31]. According to one such survey, civil servants had a higher proportion of smoking and passive smoking than other working subjects such as teachers and doctors [32]. A qualitative investigation in Shanghai also showed that alcohol-drinking behavior varied among populations [33].

We found that civil servants reported significantly better psychosocial work environments, including more social capital, lower job demand, and more support at work than participants from high-tech enterprises. However, the scores of job control were comparable. A study of Chinese civil servants reported that nearly half of respondents perceived high job stress [34]. However, another study of public servants in Macao found that only 12.93% perceived high job stress [35]. Compared with another two studies of enterprise employees and scientific and technical personnel, the mean scores of job demand were 3.43 and 3.13, which were lower than the results of the current study [35, 36]. A previous study indicated that young people had better SRH than did older subjects [37]. In our study, participants from government departments included a higher proportion of middle-aged and older subjects who reported better SRH than those from high-tech enterprises. Moreover, compared with employees from high-tech enterprises, the civil servants in our study had significantly unhealthier behaviors. This might be due the different work content and psychosocial work environment between these two types of worksite.

Based on these results, civil servants had worse lifestyles than participants from high-tech enterprises. Moreover, the proportion of male participants was higher in high-tech enterprises than in government departments. The proportion of young participants from high-tech enterprises was also higher. Several studies in Shanghai showed that males had a worse lifestyle than females, whereas young people led a worse lifestyle than middle-aged individuals [38, 39]. Therefore, it is necessary to discuss the possible reasons for these results. Civil servants are considered to have one of the most respected jobs in the eyes of the Chinese population. Because they had higher social positions than the other groups, they had more social time and opportunities for smoking and drinking than other individuals [40].

In addition, civil servants reported better a psychosocial worksite environment than participants from high-tech enterprises. The difference in the psychosocial worksite environment between government departments and high-tech enterprises was easy to understand. High-tech enterprises were perceived to have a high job demand and fast work rhythm because employees are required to update their specialized knowledge and accomplish sophisticated duties on time without error. In contrast civil servants, who are responsible for drawing up plans and providing guidance and supervision for relevant activities, are considered to have an easy job. In the current analyses civil servants had a better SRH than high-tech enterprise employees. It is possible that the psychosocial worksite environment might be more important for determining SRH than health behaviors. Although it remains unclear what is behind this interesting phenomenon, two characteristics might play a role. First, in our opinion, SRH provides a subjective assessment of one’s health, which is different from an objective health laboratory examination. In other words, subjective factors might influence an individual SRH more than objective factors. In the current study, health behaviors and psychosocial worksite environment were both measured by self-reported questionnaires rather than objective methods. However, health behaviors including physical activity, smoking, passive smoking, and alcohol drinking were reported based on participants’ objective behaviors rather than personal feelings. Personal feelings might have more effect on the measurement of a psychosocial worksite environment than health behavior. Second, determining the outcome of healthy or unhealthy behaviors will take a long period of time. For example, smokers will not realize the harmful effect of smoking until they suffer from adverse symptoms. However, the psychosocial worksite environment such as job demand and job control will affect people’s moods and feelings immediately.

Our study indicated that gender had a strong association with SRH among all participants, whereas length of service was associated with SRH in participants from high-tech enterprises when only demographic factors were included in the models.

Consistent with our findings, other studies reported that males were more likely to report good SRH than females [1, 15], and that length of service negatively correlated with the health condition of metro workers [37]. Interestingly, age and length of service were associated with SRH in different directions. Moreover, the association varied by type of worksite. For participants from high-tech enterprises, people <50 were less likely to have good SRH (OR <0.5) than participants >50. However, compared with people employed for >20 years, respondents employed for <20 years were more likely to have good SRH (OR <0.5). However, these associations were reversed in civil servants. The difference in these associations could be due to the different work content. For high-tech enterprises, employees have to update their specialized knowledge and accomplish sophisticated duties on time without error. Interestingly, younger individuals made up more of the workforce in high-tech enterprises. Considering the adverse psychosocial work environment, it is logical that employees with a shorter length of service perceived better health. For government departments, experience played an important role. Therefore, people with longer length of service worked more easily.

When lifestyle factors were introduced into the models, different factors were related to SRH after adjusting for demographic variables in the two types of worksite. Among high-tech enterprise employees, physical activity was positively correlated with good SRH significantly. However, passive smoking was negatively associated with good SRH in civil servants. Some studies also found a similar association between physical activity and SRH [17, 41]. In contrast, some studies reported that SRH was negatively associated with other health behaviors such as smoking and alcohol intake [15, 41], which was not found in our study.

Psychosocial factors were introduced into the final models. The determinants of SRH differed by type of worksite. For employees from high-tech enterprises, SRH was related to gender, education, physical activity, social capital, and job control. However, in workers from government departments SRH was associated with gender, age, passive smoking, and job control. Among the psychosocial factors, job control was positively associated with SRH in participants from both type of worksite. Consistent with our findings, other studies reported that low job control was associated with poor SRH in various working people including nurses and municipal employees [41, 42]; however, these two studies also indicated that SRH was associated with job demand and support at work, which was not found in our study. Some studies reported that social capital was related to SRH in Chinese adults [43], which partially support our findings. Social capital was positively associated with SRH in high-tech enterprises, but not among civil servants. This could be due to the high job demands and fast work rhythm of the high-tech industry, which suggested that social capital was more important for stressful job. However, improving job control was important for both stressful and relatively relaxed jobs.

In general, our results revealed some differences between the potential health-influencing factors of participants according to type of worksite in both unadjusted and adjusted models. There were different main influencing factors of health between the two work groups. For employees from high-tech enterprises, a lack of social capital and professional support should be taken seriously. If feasible, lowering employees’ job demands might be an effective way of improving their health. However, for government departments the priority should be to help civil servants establish healthy lifestyle habits, including enough physical activity and cessation of smoking. In addition to health education, environmental support, health culture, and leaders’ health behaviors play a critical role in improving civil servants’ health.

There are some limitations to our study. First, the direction of causality could not be addressed due to the cross-sectional study design. Second, all measures were based on self-reports, even though the measures have been validated [25, 26, 44]. In spite of these limitations, this study provides novel evidence regarding SRH and demographic characteristics, lifestyle, and the psychosocial work environment in China, which will be helpful for develop interventions that focus on different areas to improve employees’ health according to the type of worksite.

## Conclusions

Participants from different types of worksite reported different SRH, healthy lifestyles, and psychosocial work environment. The association between SRH and demographic characteristics, lifestyle, and psychosocial work environment differed by type of worksite significantly. Among participants from high-tech enterprises, gender, education, length of service, physical activity, social capital, and job control were associated with SRH. Among participants from government departments, gender, age, passive smoking, and job control were associated with SRH. The psychosocial work environment might be more important for determining better SRH than health behaviors among employees. It is necessary to develop interventions that focus on different areas to improve employees’ health according to the type of worksite and characteristics of the employees.

## Abbreviations

SRH: Self-rated health
IPAQ: International Physical Activity Questionnaire
SPSS: Statistical Package for Social Sciences.
